# Electrophoretic deposition of chitosan coatings on the Ti15Mo biomedical alloy from a citric acid solution

**DOI:** 10.1039/d0ra01481h

**Published:** 2020-04-01

**Authors:** Magdalena Szklarska, Bożena Łosiewicz, Grzegorz Dercz, Joanna Maszybrocka, Marzena Rams-Baron, Sebastian Stach

**Affiliations:** Institute of Materials Engineering, Faculty of Science and Technology, University of Silesia in Katowice 75 Pułku Piechoty 1A, 41-500 Chorzów Poland magdalena.szklarska@us.edu.pl; August Chełkowski Institute of Physics, Faculty of Science and Technology, University of Silesia in Katowice 75 Pułku Piechoty 1A, 41-500 Chorzów Poland; Institute of Biomedical Engineering, Faculty of Science and Technology, University of Silesia in Katowice Będzińska 39, 41-200 Sosnowiec Poland

## Abstract

Chitosan biocoatings were successfully deposited on the Ti15Mo alloy surface *via* cataphoretic deposition from a solution of 1 g dm^−3^ of chitosan in 4% (aq) citric acid. The influence of the cataphoretic deposition parameters on quality and morphology of the obtained coatings were investigated using fluorescence and scanning electron microscopy. The functional groups' presence in chitosan chine were confirmed by ATR-FTIR methods. X-ray analysis revealed the amorphous structure of the chitosan coatings on the Ti15Mo alloy surface. The conducted studies also include assessing the abrasion resistance and adhesion to the substrate of the obtained chitosan coatings. The results show that utilizing the citric acid as a solvent results in the formation of pore free coatings. The yield of the electrophoretic deposition process was in the range of 2–10 mg of deposited chitosan per 1 cm^2^. The obtained coatings through the unique properties of chitosan are a promising biomaterial for application in medicine.

## Abstract 2

Chitosan biocoatings were successfully deposited on the Ti15Mo alloy surface *via* cataphoretic deposition from a solution of 1 g dm^−3^ of chitosan in 4% (aq) citric acid.

## Graphical abstract

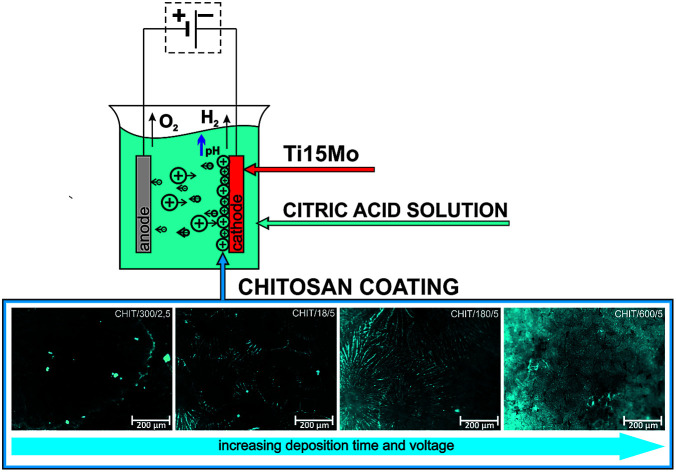

## Introduction

Implants in the human body should work as long as possible and operate flawlessly increasing thus the comfort of the patient, and extend the time between the operations necessary to replace the implant. The challenge for the present science is to obtain a biomaterial with mechanical properties similar to human bones simultaneously with a high biocompatibility and biofunctionality. A new generation of biomaterials are required to induce the desired reactions of the body or provide substances that support healing at the right time and place.^1–3^ Metallic materials are the most commonly used materials in implantology.^4^ The research to date shows that the possibilities of optimization of mechanical properties and biocompatibility of metallic materials *via* selection of phase and chemical composition as well as heat and plastic treatment are largely exhausted.^5^ Therefore, currently work is being carried out on the development of technology for surface modification of metallic materials by applying thin layers or coatings. In order to increase the biocompatibility of implants the oxide layers, polymeric or ceramic coatings are applied on their surface.^6–12^ They increase the corrosion resistance of the implant and may also be a matrix with embedded tissue-forming, antibacterial and/or anticoagulant substances.^13^ An interesting technique for obtaining coatings for medical use is the electrophoretic deposition (EPD) method.

**Fig. 1:**
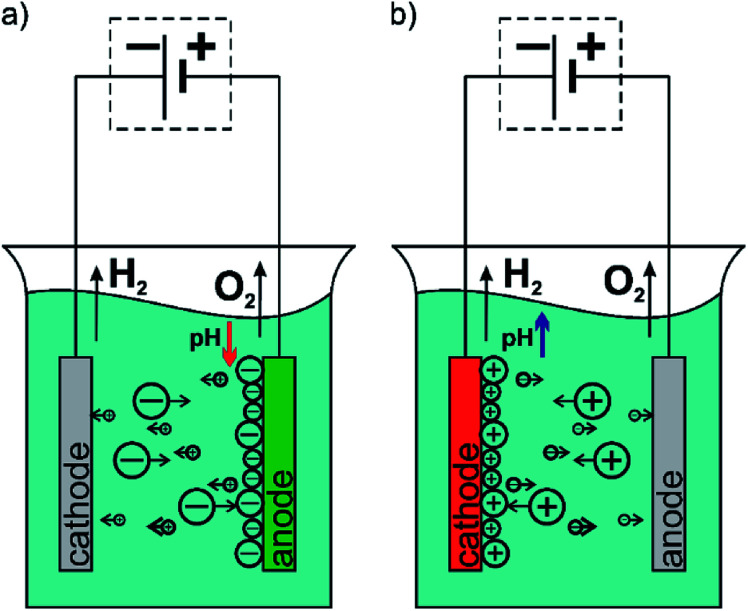
Diagram of the electrophoretic deposition process: (a) anaphoretic deposition and (b) cathaphoretic deposition.

The EPD method is a universal technique to deposit great variety of materials such as metals, ceramics, glasses, polymers and their composites.^14^ Moreover, the EPD could be applied both to simple, small devices as well as those large with complex shape. This method does not require high-tech equipment and it is cost-effective. The EPD owes its great popularity to the ability to easy control of the thickness and morphology of formed coatings by setting appropriate parameters of the process.^15^ The EPD is carried out in two-electrode cell and is based on the phenomenon of electrophoresis, *i.e.* the motion of charged particles in the solution toward electrode under applied electric field. Based on this it could be distinguish two types of the EPD *via* an anaphoretic deposition when deposition of negative charged particles occurs at anode (Fig. 1a) and cathaphoretic deposition when the coatings are formed on the cathode surface (Fig. 1b).^15^

Recently, the EPD is widely used to obtain biofunctional coatings on the implant surface.^11,14^ Especially, chitosan (CHIT) coatings are of great interest. Chitosan is a cationic linear polysaccharide, which is valued by medicine because of its unique properties. It is worth to mention that chitosan have antifungal and antimicrobial properties.^8,11,16–19^ Furthermore, chitosan is biodegradable and non-toxic for human body, and additionally promotes regeneration of the surrounding tissues.^11,20^ Due to good adhesion with both soft and hard tissues, chitosan has been used in dentistry, orthopaedics, ophthalmology and surgery.^21^ Because it is a cationic polymer, it can form polyelectrolyte complexes with negatively charged biomolecules, which is used in controlled drug delivery systems.^22^

In this work, the surface modification *via* EPD of chitosan coatings was carried out to improve properties of the Ti15Mo alloy as a biomaterial. This alloy is interesting and promising material to use in bone implantology because of its mechanical properties similar to humane bones and high corrosion resistance in environment of human body.^23–26^ The Young's modulus of the Ti15Mo alloy (77 GPa), most closely related to the Young's module of the bone (15–25 GPa), is about 30% smaller than for pure titanium or Ti6Al7Nb alloy and as much as 60% smaller than Young's modulus of 316L implanted steel.^27^ What is more, alloying elements, such as Ti and Mo do not show toxicity to living organisms.^28–31^ The Ti15Mo alloy due to the above-mentioned properties is a potential material for the production of long- and short-term bone implants, such as joint endoprostheses, surgical devices and medical device components. In the production of implants, in addition to suitably selected material properties, it is very important to modify their surface, ensuring proper connection and interaction at the implant–tissue interface.

The main purpose of this work was to obtain chitosan coatings on the Ti15Mo alloy surface *via* electrophoretic deposition and its chemical and physical characterization in dependence of the EPD parameters.

## Materials and methods

### Material preparation

Chitosan powder (Sigma-Aldrich) (1 g dm^−3^) with medium molecular weight (MW = 80 kDa) and deacetylation degree of 75–85% was dissolved in a 4% (v/v) aqueous solution of the citric acid. The solution was prepared using nanopure water (Milli-Q, resistivity of 18.2 MΩ cm) and magnetically stirred for 24 h to ensure complete dissolution of chitosan.

The tested samples of the Ti15Mo alloy in shape of disc were cut from the rod of 10 mm in diameter and mechanically polished with 600 to 5000# grit silicon carbide paper. Then they were polished using Struers Md-Mol polishing cloths and OP-S polishing suspension. The samples were ultrasonically cleaned with nanopure water and acetone. Just before the EPD, the electrodes were depassivated for 1 min in a solution containing H_2_SO_4_ (4 mol dm^−3^) and HF (1 mol dm^−3^), and then rinsed with nanopure water.

For the EPD process the electrochemical cell with two parallel electrodes placed in distance of 1.5 cm was used. The Ti15Mo alloy was the working electrode and the platinum foil constituted as a counter electrode. Deposition of chitosan coatings was conducted using MAG-5N galvanizing aggregate at deposition voltage of *U* = 2.5–10 V for time of deposition of *t* = 18–600 s at 25 °C (Table 1). The deposited chitosan coatings were carefully rinsed with nanopure water, dried at room temperature for 24 h and weighted.

**Table 1:** Summary of the EPD parameters for chitosan coatings on the Ti15Mo alloy at 25 °C

Coating name	Electrolyte	Time/s	Voltage/V
CHIT/18/5	4% (aq.) citric acid + 1 g dm^−3^ of CHIT	18	5
CHIT/60/5		60	5
CHIT/180/5		180	5
CHIT/300/5		300	5
CHIT/600/5		600	5
CHIT/300/2.5		300	2.5
CHIT/300/7.5		300	7.5
CHIT/300/10		300	10

## Material characterization

The X-ray diffraction (XRD) measurements were performed on the X'Pert Philips PW 3040/60 diffractometer operating at *I* = 30 mA and *U* = 40 kV. The wavelength of radiation (*λ* Cu K_α_) was 1.54178 Å. The grazing incidence X-ray diffraction (GIXD) patterns were registered in the 2*θ* range from 10 to 80°.

Attenuated total reflectance Fourier transform infrared (ATR-FTIR) spectroscopy (Schimadzu IR Prestige-21) was used for determination the functional groups of the obtaining chitosan coatings.

The microstructure of the obtained chitosan coatings were investigated using IX81 inverted Olympus microscope equipped with filter CFP and JEOL JSM-6480 scanning electron microscope (SEM).

The surface roughness of the tested biomaterials was determined using the Mitutoyo Surftest SJ-500/P profilometer. Changes in the surface profile were measured with a measuring step of 0.1 μm and a speed of 200 μm s^−1^, over a length of about 10 mm.

The OLYMPUS LEXT OLS4000 confocal laser microscope (CLM) was used to determine the thickness of the obtained chitosan coatings. The measurement consisted of scanning the surface area of the Ti15Mo alloy partially covered with the polymer coating, and then 3D reconstructing of this surface area, obtaining a geometrical structure of the test surface (SGS). A series of profiles from the interface between the substrate and the polymer coating has been designated on the obtained surface map. Analysis of the height difference between the surface of the chitosan coating and the surface of the substrate allowed to determine the thickness of the deposited polymer layer.

The tests of tribological properties of the obtained chitosan coatings were aimed at determining their abrasion resistance and adhesion to the substrate. Tribological tests in the reciprocating motion were conducted using the Anton-Paar tribometer in the ball-flat type. The test was carried out under a 1 N load with an average speed of 2 cm s^−1^ by a 4 mm friction path. The counter-sample was a ZrO_2_ ball with a diameter of 6 mm. The tests were carried out under dry friction conditions. The test parameters were selected after preliminary study. The wear of the obtained materials after tribological tests was determined on the basis of microscopic observations. Observation of the surface was carried out using an OLYMPUS optical GX-51 microscope and the MOOL JSM-6480 scanning electron microscope.

The scratch test was used to assess the strength of the adhesion of polymeric coatings with the substrate and was carried out using CSM Instruments micro scratch tester on compact platform with an integrated microscope. The test consisted of a controlled crack on the surface of the Ti15Mo alloy with a polymer coating applied using a Rockwell diamond indenter with a radius of 100 μm. The indenter load increased at a constant speed of 1 to 1000 mN at a distance of 3 mm and a travel speed of 6 mm min^−1^. Observations of scratches were carried out using an optical microscope coupled to the device. The optical analysis combined with the registered characteristics allowed to determine the critical loads, *L*_c_, the smallest force at which the coating was damaged.

## Results and discussion

### Chitosan coatings characterization

**Fig. 2:**
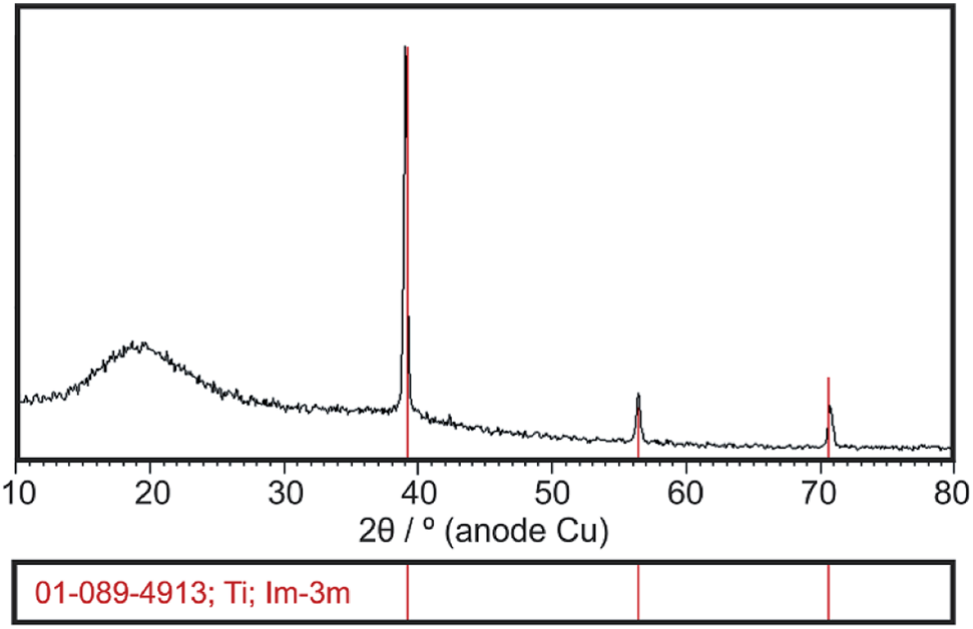
Exemplary XRD pattern of the chitosan coating deposited at 5 V for 5 min on the Ti15Mo alloy.

XRD analysis confirmed the presence of amorphous state of deposited chitosan coatings which is evidence by the amorphous halo broad reflection at 2*θ* = 20 (Fig. 2). The XRD pattern shows also diffraction lines of β-phase titanium (ICDD PDF 01-089-4913) originated from the Ti15Mo substrate.

**Fig. 3:**
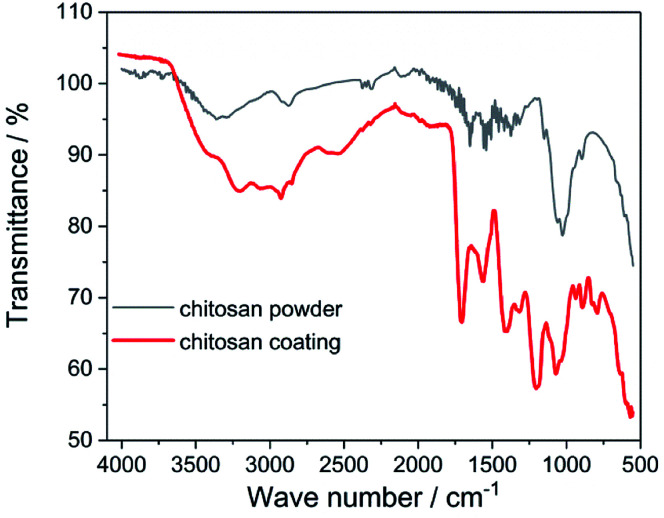
ATR-FTIR spectrum of the chitosan powder and coating deposited at 5 V for 5 min on the Ti15Mo alloy.

ATR-FTIR studies allowed to determine the functional groups characteristic for the chitosan (Fig. 3). The peaks located at 2924 cm^−1^, 1467 cm^−1^ and 1317 cm^−1^ are assigned to the stretching vibration of the methylene group –CH_2_, present in the typical for saccharides pyranose ring.^32–35^ Glycosidic bonding –C–O–C– occurring between repeating unit form CH gives characteristic bands around 1151 cm^−1^ and 894 cm^−1^.^11,33,36^ The effect of the C–O stretching vibrations are bands at 1029 cm^−1^ and 1074 cm^−1^.^11,33,35,36^ In the FTIR spectra for both chitosan powder and coating there are a presence of the band at 1651 cm^−1^ derived from stretching vibration of C=O amide group (amide I band), band at 1573 cm^−1^ corresponding to the covalent vibration of N–H at –NH_2_ group and band at 1558 cm^−1^ which can be attributed to bending vibrations of the NH group of the amide.^11,33–39^ In the FTIR spectrum registered for the chitosan coating, the additional bands at 1713 cm^−1^, 1401 cm^−1^ and 1200 cm^−1^ resulting from the vibrations of C=O, C–O and C–O–C bonds derived from citric acid, are visible.^40–42^ These results indicate that on the Ti15Mo alloy surface besides chitosan, also citric acid molecules were deposited.

**Fig. 4:**
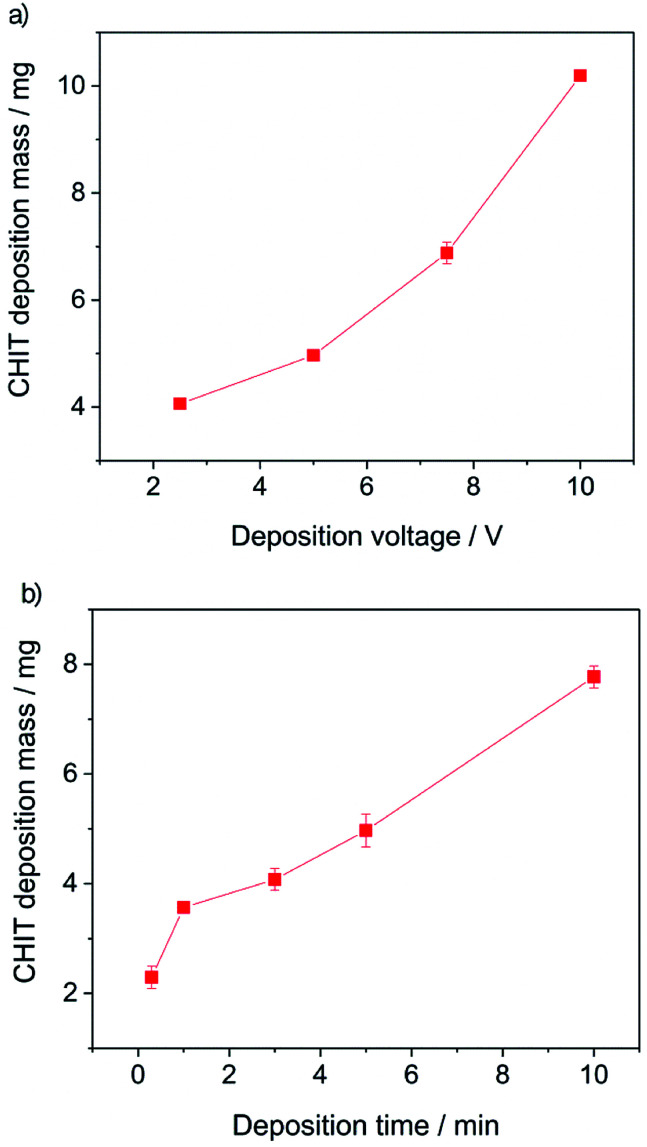
Chitosan deposition mass on the Ti15Mo alloy as a function of: (a) deposition time at constant voltage (5 V), and (b) deposition voltage at a constant time (300 s) from a solution of 1 g dm^−3^ of chitosan in 4% (aq) citric acid.

Further analysis of the obtained results allowed to determine the influence of the EPD process parameters on the efficiency of the deposition process (Fig. 4).

From the obtained results, it can be concluded that both the increase of the deposition time and the deposition voltage results in an increase in the mass (amount) of the deposited polymer and the thickness of the chitosan coating on the Ti15Mo alloy. The yield of the EPD process carried out from a 4% aqueous solution of citric acid is 2–10 mg cm^−2^. The EPD of chitosan coatings is possible due to the presence of positively charged polymer molecules in solution. Chitosan is dissolved in dilute acids according to the reaction:

1 CHIT–NH_2_ + H_3_O^+^ → CHIT–NH_3_^+^ + H_2_O

and the amino group in the polymer chain are protonated. In the EPD process, the applied electric field provides electrophoretic movement of positively charged chitosan molecules towards the cathode (Fig. 1b). Simultaneously the pH increases around the cathode surface due to the electrolytic decomposition of water molecules:

2 2H_2_O + 2e^−^ → H_2_ + 2OH^−^

As a result of the neutralization of the protonated amino groups of the chitosan chain in the presence of OH^−^ ions, an insoluble chitosan coating is formed on the cathode surface:^14^

3 CHIT–NH_3_^+^ + OH^−^ → CHIT–NH_2_ + H_2_O.

**Fig. 5:**
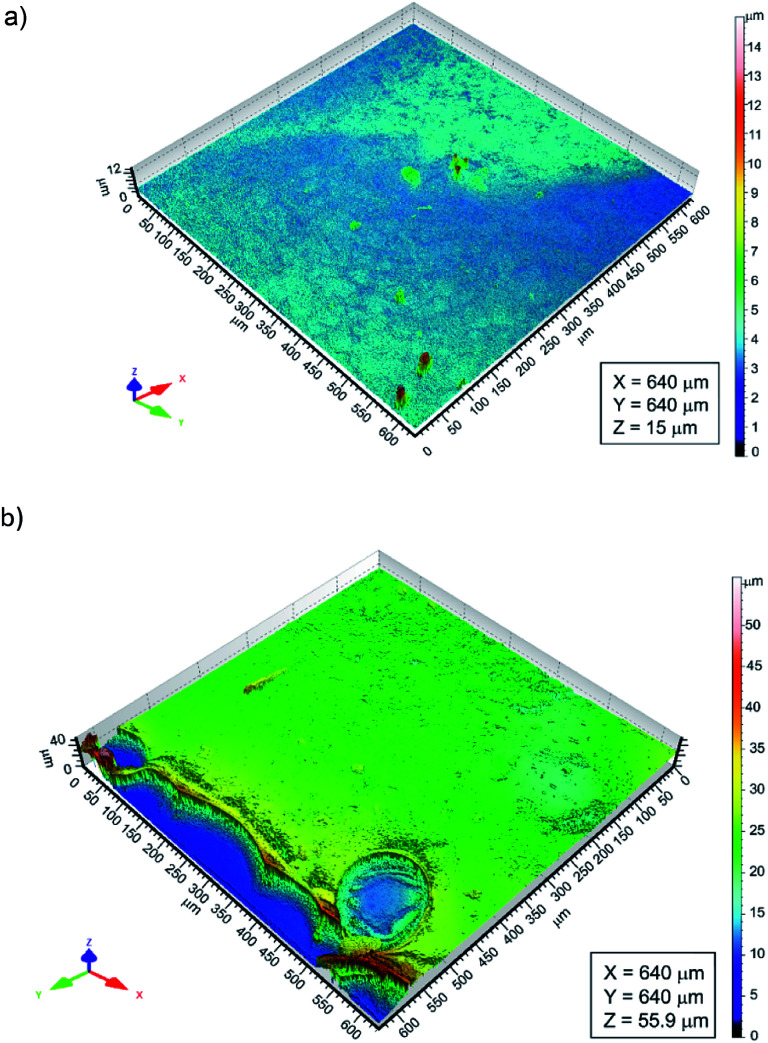
The surface topography map from the area containing the boundary between the coating and the substrate, registered with a confocal laser microscopy for chitosan coatings deposited on the Ti15Mo alloy surface at: (a) 5 V for 60 s and (b) 7.5 V for 300 s.

The thickness of the chitosan coatings deposited on the Ti15Mo substrate was determined using a confocal laser microscope. The CLM observations comprised areas of the Ti15Mo alloy surface on which the substrate itself and the applied chitosan coating were visible (Fig. 5). Determining differences in height between the surface of the substrate and the surface of the polymer film, the thickness of the deposited chitosan coating was determined. It ranged from 0.81 to 15.87 μm.

**Fig. 6:**
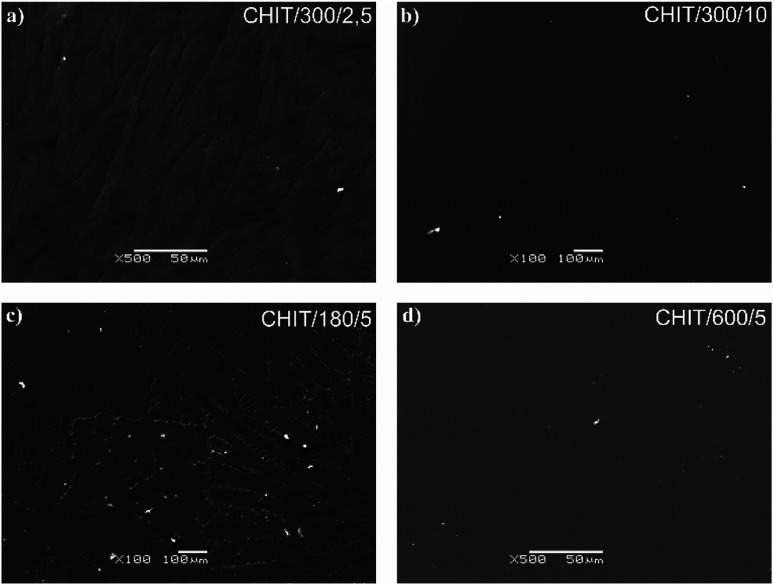
SEM images of the chitosan coatings deposited *via* EPD on the Ti15Mo alloy at different time/voltage: (a) 300 s/2.5 V, (b) 18 s/5 V, (c) 180 s/5 V, and (d) 600 s/5 V.

**Fig. 7:**
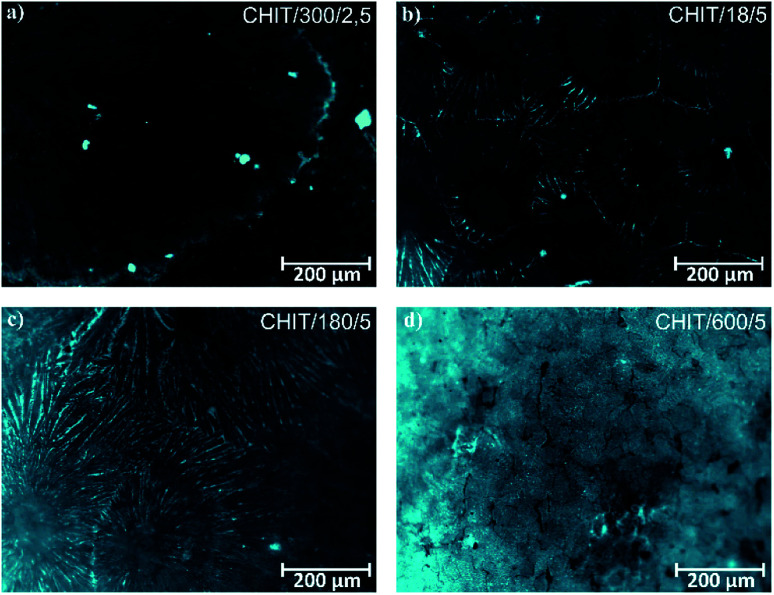
Fluorescence microscopic images of the chitosan coatings deposited *via* EPD on the Ti15Mo alloy at different time/voltage: (a) 300 s/2.5 V, (b) 18 s/5 V, (c) 180 s/5 V, and (d) 600 s/5 V.

Variation of deposition time and voltage have also influence on the morphology of the formed chitosan coatings. Microscopic analysis revealed that with extending time of the EPD the obtained chitosan coatings were smoother and more homogeneous (Fig. 6). Short deposition time, below 180 s, results in formation of the chitosan coatings with dendritic structure resembling flowers (Fig. 6c, Fig. 7b and c). From the centre of the flower structure the dendritic chitosan bands are concentrically spread. Observations at high magnification allow to state that the thin chitosan coating deposited for a short time are rough and that the roughness disappears with increasing of the deposition time. Moreover, based on the luminescent intensity which depends on the amount of the deposited chitosan it could be concluded that elongation of the EPD process leads to the thicker coatings formation (Fig. 7). Similarly the deposition voltage allows to control the amount of the deposited chitosan and the thickness of the coatings (Fig. 6). At low deposition voltage of 2.5 V the dendritic formation with multiple branches could be observed (Fig. 6a and 7a). Thick, dense and homogenous chitosan coatings were obtained at 5 and 10 V for 600 and 300 s, respectively (Fig. 7). What is interesting utilizing 4% citric acid with pH 2 as a solvent results in formation of the porous-free chitosan coatings. The formation of pores in the chitosan coating is related to the rate of the coating deposition and the intensity of hydrogen evolution at the cathode due to the electrolytic decomposition of water. The deposition rate depends on the deposition voltage but also on the pH of the EPD solution. Chitosan is soluble in diluted acids below ∼ pH 5. Simchi and co-workers^20^ stated that lowering the pH of the solution increases the amount of protonated amino groups and thus the ionization of the solution. A large number of protonated amino groups in the polymer chain slows down the process of its neutralization at the cathode surface, resulting in relatively slow deposition rate of the insoluble chitosan. In this study the low pH of the chitosan solution in the 4% citric acid (pH = 2) resulted in a decrease in the deposition rate. Therefore, when the growth rate is relatively low, the forming chitosan coating is not able to entrap the evolving hydrogen gas and a dense chitosan film is formed.

**Fig. 8:**
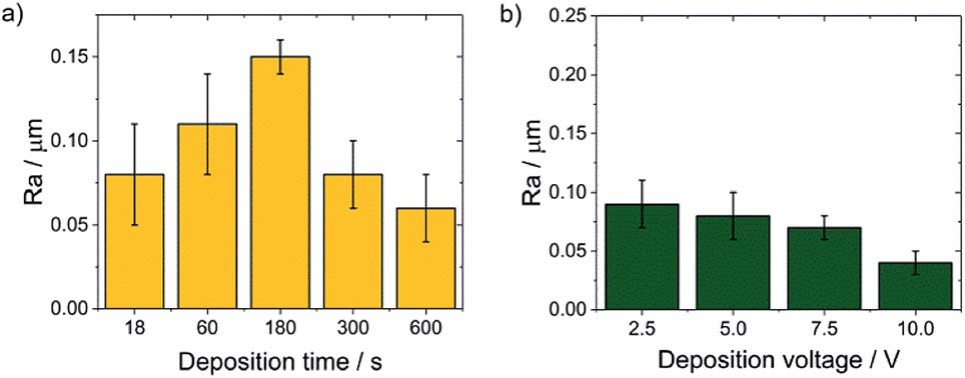
The dependence of the *R*a value on the EPD parameters for the CHIT coatings deposited on the Ti15Mo alloy at: (a) constant voltage value of 5 V and (b) constant time of 300 s.

SEM observations showed that the morphology of the obtained chitosan coatings strongly depends on the EPD parameters, therefore the next stage of the research was to determine the roughness of the deposited coatings from a natural biopolymer in the form of chitosan. The results of the roughness study of the obtained chitosan coatings are in good agreement with microscopic observations. The obtained results indicate that along with the deposition time from 18 to 180 s at a constant voltage of 5 V, the value of the arithmetical mean deviation, *R*a increases from 0,08(3) to the value of 0.15(1) μm (Fig. 8a). These results coincide with microscopic observations showing the dendritic structure of the chitosan coatings deposited at a such process parameters. With increasing deposition time up to 600 s, the dendritic structure disappears and a smooth, homogeneous chitosan coating begins to form, which is reflected in the roughness results. The *R*a value decreases to 0.08(2) μm and 0.04(1) μm for 300 s and 600 s of deposition time, respectively. Higher deposition voltages also favor the formation of smooth and homogeneous chitosan coatings. The lowest value of the *R*a = 0.04 μm was observed for the chitosan coating deposited at 10 V for 300 s (Fig. 8b).

### Assessment of wear mechanisms

Both the Ti15Mo alloy in the initial state as well as the Ti15Mo alloy covered with the chitosan coatings were subjected to tribological tests (Table 2).

**Table 2:** Comparison of the results obtained during tribological test of the chitosan coatings deposited *via* EPD on the Ti15Mo alloy surface (see Table 1)

Coating name	Friction distance/m	Cycles	Kinetic friction coefficient	Wear width/μm
CHIT/300/5	1.10(1)	138(1)	0.159(5)	124(3)
CHIT/600/5	1.02(3)	128(1)	0.152(3)	319(1)
CHIT/300/7.5	3.59(5)	449(5)	0.186(4)	207(5)
CHIT/300/10	8.82(3)	1100(5)	0.189(1)	117(4)

Determination of the friction coefficient for the Ti15Mo alloy in the initial state (*μ* = 1.35) allowed in the subsequent tests clearly determine the moment of abrasion of the chitosan coating and reaching the ground material. Thin chitosan coatings deposited at low voltages (less than 5 V) and in a short time (below 5 min), subjected to tribological tests at a given load of 1 N, were immediately abraded. The kinetic friction coefficient determined for thicker chitosan coatings was in the range from 0.152(3) to 0.189(1), and the most durable of the chitosan coatings deposited at 10 V for 5 min, rubs after 1100 cycles which corresponds to a friction distance equal 8.82(3) m.

**Fig. 9:**
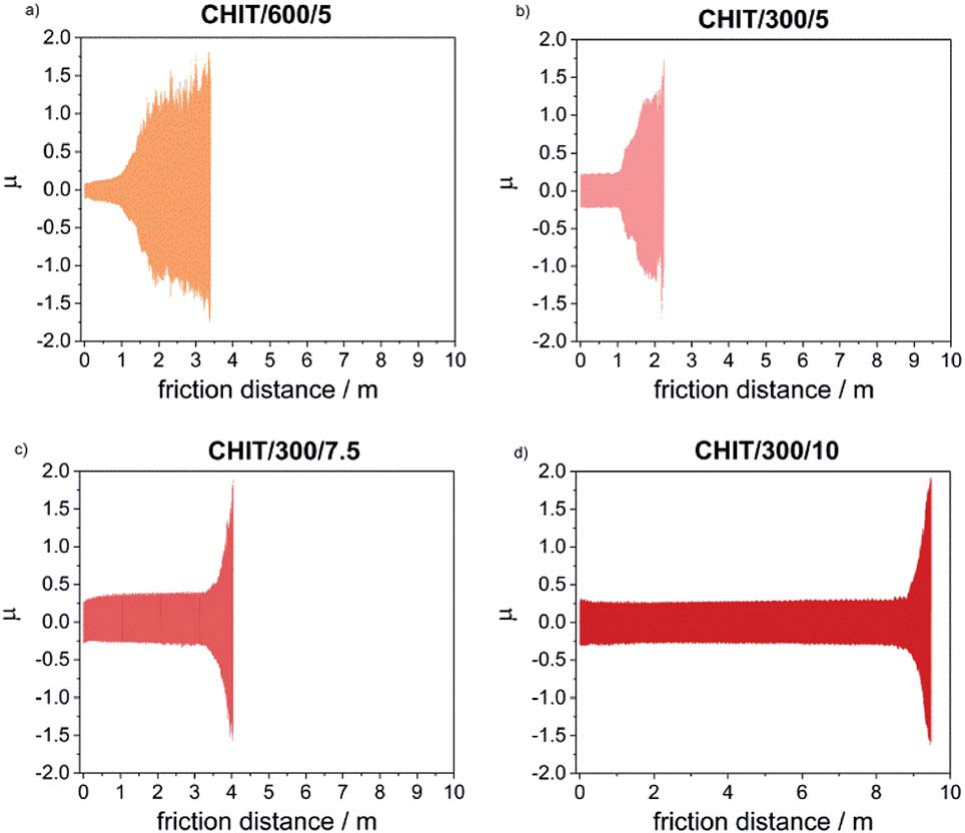
Changes in the friction coefficient registered during tribological tests of the Ti15Mo alloy covered with the CHIT coatings deposited on its surface *via* EPD at different time/voltage parameters: (a) 600 s/5 V, (b) 300 s/5 V, (c) 300 s/7.5 V and (d) 300 s/10 V.

With the reduction of the deposition voltage, the abrasion resistance of the obtained chitosan coatings decreased (Table 2, Fig. 9). Reducing the deposition voltage results in formation of chitosan coatings with lower abrasion resistance. The results obtained for the chitosan coatings deposited at the same voltage of 5 V but for different time of 5 and 10 min, were slightly differed from each other.

**Fig. 10:**
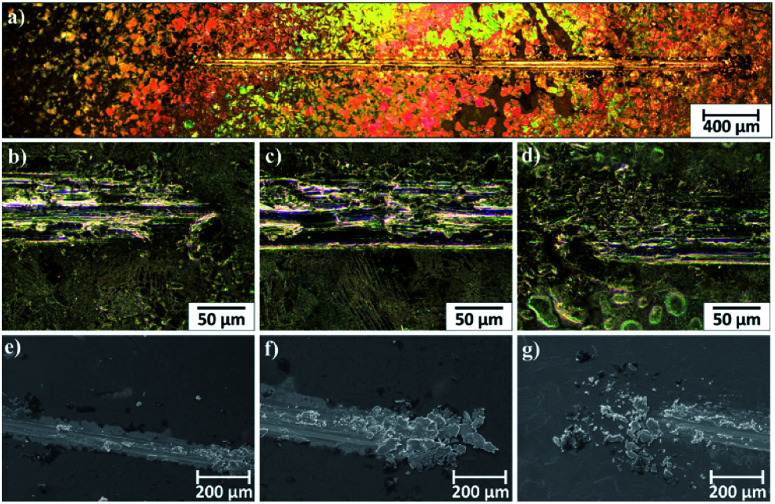
Area of wear track for the CHIT/300/5 coating on the Ti15Mo alloy observed using light microscopy: (a) in light field and (b–d) in dark field and (e–g) SEM.

**Fig. 11:**
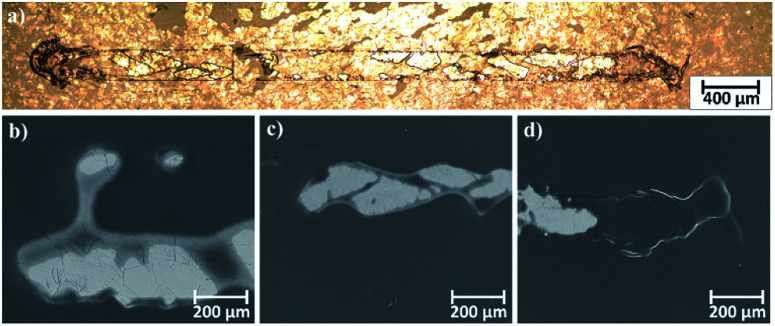
Area of wear track for the CHIT/600/5 coating on the Ti15Mo alloy observed using: (a) light microscopy in light field and (b–d) SEM.

**Fig. 12:**
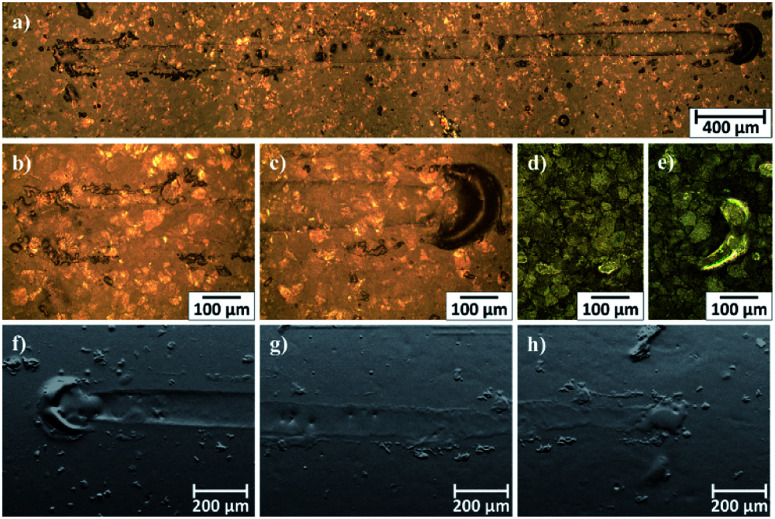
Area of wear track for the CHIT/300/7.5 coating on the Ti15Mo alloy observed using light microscopy: (a–c) in light field and (d and e) in dark field and (e–g) SEM-BSE.

Microscopic observations of wear track allowed to asses mechanical wear. For the CHIT/300/5 coating there is clear wear track with visible products of wear at its ends. Flaked fragments of this coating are also visible on the edges of wear track (Fig. 10). For the CHIT/600/5 coating there is visible fraction trace in chitosan film (Fig. 11). At the end of the friction trace a thickened and corrugated crescent-shapes polymer coating is observed. That indicates that the coating material has been pushed and pressed by the indenter. Within the fraction trace, areas in which the chitosan coating was peeled off, were observed. These fragments are clearly visible in SEM images, where bright areas are places where the polymer coating has been torn off and the structure of the substrate is visible (Fig. 11b and d). In these areas, grains characteristic for the substrate material are observed. It is a case of a mixed wear mechanism, including adhesive and fatigue mechanism.^43^ The chitosan coating material, as a result of cyclic deformation was delaminated, peeled off and removed from the friction zone. For chitosan coatings deposited for 5 min at higher deposition voltages of 7.5 and 10 V, a trace of abrasion in the coating is observed without visible tear of polymer film to the substrate (Fig. 12). In this case the deformation and fatigue wear mechanism occurs.^43^ At the ends and edges of the friction trace, there are characteristic for chitosan-coated samples, the thickening of polymer film which are forming directly in front of the indenter. At the edges of the friction trace, there are also a few wear products in the form of small exfoliations of the chitosan coating. SEM observations also have not shown clear abrasion in the chitosan coating. Nevertheless, a rapid change in the kinetic friction coefficient indicates a tribological response from the alloy substrate.

**Fig. 13:**
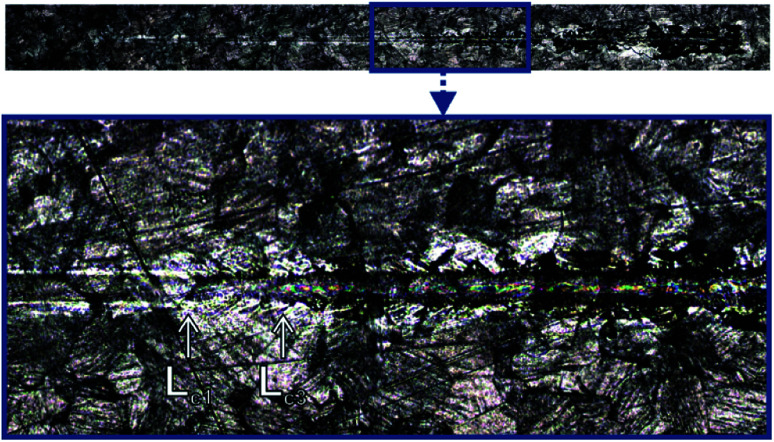
The scratch trace of the CHIT coating deposited *via* EPD on the Ti15Mo alloy surface at 5 V for 600 s.

The scratch test was carried out for the CHIT/600/5 coating. Two critical loads, *L*_c1_ = 521.23(9) mN and *L*_c3_ = 546.01(23) mN, were registered during the test (Fig. 13). After exceeding the first critical load, *L*_c1_ the first cracks of the coating start appears. The cracks are perpendicular to the direction of the indenter movement and have the character of cohesive cracks. Complete abrasion to the substrate and large peelings and chipping of the chitosan coating are observed above the load *L*_c3_.^44^

## Conclusion

The conducted studies confirm possibility to obtain the amorphous chitosan coatings on the Ti15Mo alloy *via* EPD. What is more by the controlling of the deposition parameters, namely time and voltage, the thickness and morphology of the chitosan coatings can be tailored. Utilization of citric acid as a solvent in electrochemical bath results in a homogeneous pore-free chitosan coatings. The thickest and the most smooth chitosan coatings was obtained at deposition voltage 10 V for 300 s and 5 V for 600 s. The tribological analysis shows that thicker chitosan coatings have better abrasion resistance. In the case of chitosan coatings deposited at low voltages (5 V) the mixed wear mechanism, including adhesive and fatigue mechanism occurs. Moreover, large abrasions to the substrate are observed. For the coatings deposited at 7 V and higher the deformation and fatigue wear mechanism take place.

The carried out studies encourage further research on the surfaces modification of titanium alloys *via* EPD of coatings from natural polymer like chitosan. The obtained bioresorbable chitosan coatings can serve as a matrix immobilizing bioactive nanoparticles, tissue-producing substances and drugs supporting the adoption of an implant in the human body. Acquired knowledge about the mechanism of EPD process, the impact of the deposition process parameters and the composition of the deposition solution will facilitate the future development of hybrid and composite coatings based on chitosan.

## Conflicts of interest

There are no conflicts to declare.

## Supplementary Material
